# The use of the terrestrial snails of the genera *Megalobulimus* and *Thaumastus* as representatives of the atmospheric carbon reservoir

**DOI:** 10.1038/srep27395

**Published:** 2016-06-08

**Authors:** Kita D. Macario, Eduardo Q. Alves, Carla Carvalho, Fabiana M. Oliveira, Christopher Bronk Ramsey, David Chivall, Rosa Souza, Luiz Ricardo L. Simone, Daniel C. Cavallari

**Affiliations:** 1 Laboratório de Radiocarbono, Universidade Federal Fluminense, Av. Gal. Milton Tavares de Souza, s/n, Niterói, 24210-346, RJ, Brazil; 2 Oxford Radiocarbon Accelerator Unit, University of Oxford, Dyson Perrins Building, South Parks Road, Oxford, OX1 3QY, United Kingdom; 3 Departamento de Geoquímica, Universidade Federal Fluminense, Outeiro São João Batista, s/n, Niterói, 24001-970, RJ, Brazil; 4 Departamento de Biologia Marinha, Universidade Federal Fluminense, Outeiro São João Batista, s/n, Niterói, 24001-970, RJ, Brazil; 5 Museu de Zoologia da Universidade de São Paulo, Avenida Nazaré, 481, São Paulo, 04263-000, SP, Brazil

## Abstract

In Brazilian archaeological shellmounds, many species of land snails are found abundantly distributed throughout the occupational layers, forming a contextualized set of samples within the sites and offering a potential alternative to the use of charcoal for radiocarbon dating analyses. In order to confirm the effectiveness of this alternative, one needs to prove that the mollusk shells reflect the atmospheric carbon isotopic concentration in the same way charcoal does. In this study, 18 terrestrial mollusk shells with known collection dates from 1948 to 2004 AD, around the nuclear bombs period, were radiocarbon dated. The obtained dates fit the SH1-2 bomb curve within less than 15 years range, showing that certain species from the *Thaumastus* and *Megalobulimus* genera are reliable representatives of the atmospheric carbon isotopic ratio and can, therefore, be used to date archaeological sites in South America.

Archaeological shellmounds are the testimony of the hunter-fisher-gatherer populations who first occupied the Brazilian coast. They are dated mostly from 5 to 2 ka BP and can be found along a considerable extension of the Brazilian coastline^1^. The archaeological remains usually recovered in such sites are charcoal fragments (scattered or associated with hearths), human bones, lithic tools (e.g. scrapers, mortar and pestles), stone arrow points and a great amount of food remains such as animal bones, mollusk shells (both marine and terrestrial) and fish otoliths. More than simple remnants, the shells constitute the base of the mounds, which can reach up to 30 m in height. Despite being the most abundant and well preserved material found in this type of site, for chronological studies marine shells are often avoided due to the lack of knowledge about local marine reservoir effects^2^. Therefore, charcoal has become the most commonly dated material by archaeologists^3^,^4^.

Charcoal dating has to be performed with care due to the possibility of the old wood effect, that is the use of long-lived trees in fires, which would reflect the age of the wood instead of the time of the burning^5^. However, the anthracological analysis of the samples can prevent the use of such species or otherwise allow recognizing charcoal from barks or twigs, which would be contemporary of the death of the tree^6^. Moreover, depending on the way charcoal samples are found over the settlement, different information can be achieved^7^. Concentrated charcoal, usually associated with hearths, is more visible in the excavations, but there is also a great amount of scattered charcoal present in the sediment^8^,^9^,^10^,^11^. The latter is more difficult to be contextualized within the site due to possible events of bioturbation and weathering processes that would disturb its archaeostratigraphy.

In this context, the use of terrestrial mollusk shells as representatives of the atmospheric carbon isotopic ratio presents itself as a possibility of dating more and better distributed and contextualized samples over the settlement. However, as with charcoal, care should be taken in choosing the appropriate species for sampling. Some terrestrial mollusks scrape carbonate from limestone, which is a source of old carbon, i.e. low in radiocarbon content. This depleted carbon 14 material can be incorporated into the animal shell and result in false old dates^12^,^13^,^14^,^15^,^16^,^17^,^18^,^19^. Goodfriend & Hood^13^ reported 0–33% dead carbon incorporation in gastropod shells from Jamaica in regions with limestone. Dye^15^ compared gastropod shells from Pleistocene limestone and Holocene volcanic coasts with known collecting dates and estimated apparent ages of up to 620 yr in the former for shells of the same species. Quarta *et al.*^16^ studied two gastropod species from Italy and found an age anomaly of 1000 yr.

Goodfriend & Hood^13^ describe the different pathways by which limestone carbon can end up as shell carbonate. Yates^20^ discussed the incorporation of old carbon by diagenesis that would add up to the limestone effect. Stott^19^, based on a labelled feeding experiment, suggested that only metabolic carbon would influence the isotopic composition of the shell. Despite the limestone effect, in other works, some species were found to be in equilibrium with the atmosphere during the animal’s life and therefore would provide reliable dates^21^,^22^. Pigati, Rech & Nekola^21^ studied 46 different species of land snails from habitats on carbonate terrains. Amongst these, 78% did not contain old carbon, and only 3% contained more than 10% dead carbon. Therefore, there may be differences related to carbonate incorporation between species. Also, for fossil shells of small terrestrial gastropods recovered from well-dated, late-Pleistocene sediments in the Midwest of the United States, they found ages that were statistically indistinguishable from ages obtained from well-preserved plant macrofossils (wood, bark, plant remains)^21^. Carvalho *et al.*^22^ compared archaeological charcoal and land snails from the same context and have shown that the terrestrial mollusk *Thaumastus achilles* (Pfeiffer, 1852) provides reliable atmospheric dates. In such work those dates were used for marine reservoir effect calculations.

In the present study, the choice of samples belonging to the genera *Megalobulimus* and *Thaumastus* (Fig. 1), out of the hundreds of terrestrial snails genera present in South America^23^, was based on their common occurrence in the region, both at present and within archaeological context. Both genera are widespread, but endemic in South America, where they are easily found, allowing comparative studies in other areas of the continent.

In many of the Brazilian shellmounds, terrestrial mollusk shells are widely distributed. In the sites located on the Rio de Janeiro coast, specimens of *Thaumastus* sp. and *Megalobulimus* sp. are abundant. Therefore, the use of these snails as archaeological records for dating purposes could be an important tool in the study of hunter-fisher-gatherers settlements in Brazil. The aim of this study is to evaluate the use of such species as representatives of the atmospheric carbon reservoir.

Radiocarbon concentration in the atmosphere varies over time mainly due to variations in Earth magnetic field and solar activity. Provided that an organism is in isotopic equilibrium with the atmospheric reservoir, its initial concentration is easily related to the atmospheric calibration curves and reliable dating can be performed.

Due to nuclear weapons tests carried out by different countries, the ^14^C atmospheric concentration started rapidly increasing in 1955 AD reaching a maximum in the 1960s^24^. This so-called bomb peak was higher in the Northern Hemisphere, since this is where most of the tests were performed^25^, and almost doubled the quantity of ^14^C in terrestrial materials^26^,^27^,^28^. Since then, the atmospheric levels of bomb ^14^C have decreased due to the incorporation of this carbon into the global carbon cycle and the industrial effect acting in the opposite direction^24^,^29^,^30^,^31^,^32^. Currently, atmospheric ^14^C is slightly higher than its pre-bomb value.

The difference in bomb ^14^C atmospheric concentration between the Hemispheres generated a great ^14^C gradient in the troposphere (between North and South, and between high and low latitudes) during the early bomb period. Consequently, the contrast in ^14^C content between regional tropospheric air masses was increased and excess ^14^C was transferred southwards. Atmospheric circulation drove the spatial distribution of bomb ^14^C in the troposphere during the early bomb period^33^,^34^,^35^. This does not create a simply latitudinal gradient of ^14^C, but 3 different zones in the Northern Hemisphere and 2 zones in the Southern Hemisphere. Brazilian south, southeast, and part of west-central and northeast regions belong to zone 1–2 (Fig. 2), therefore the post-bomb atmospheric SH1-2 calibration curve^35^ should reflect the isotopic composition of terrestrial snails’ shells from this region since 1950 AD (Fig. 3). The curve is based on annual data but using mean values for summer months, necessary to produce unbiased weighted zonal mean values. According to Hua, Barbetti & Rakowski^35^, SH zone 1–2 was compiled using a separate dataset from zone 3 in the period from 1950 to 1972, and for the period from 1973 onwards, only 1 dataset for the SH was compiled.

In order to evaluate carbon isotopic ratios in terrestrial mollusk shells, we have dated 18 individual snail shells from the repository of the Zoology Museum of São Paulo University (MZUSP) which were collected around the radiocarbon bomb peak period, between 1948 and 2004 AD.

## Results

**Figure 2:**
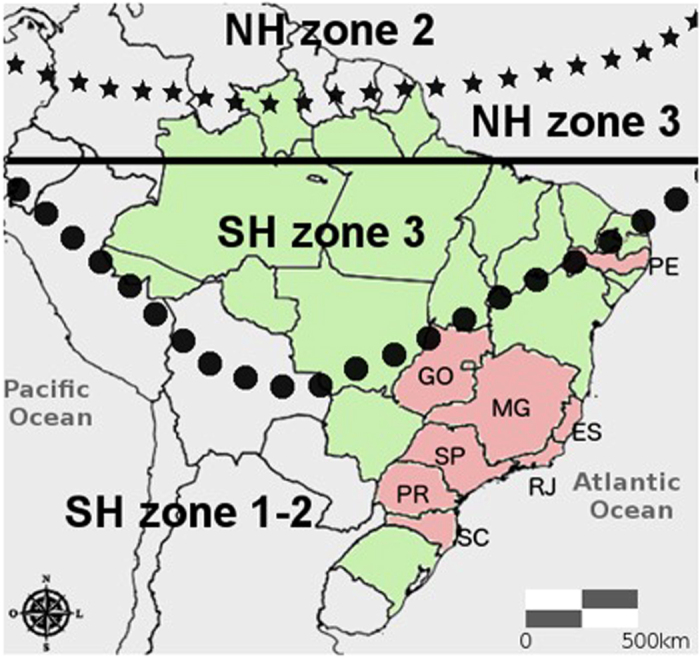
South America map showing the different zones considered by Hua *et al.*^35^ and Brazilian states where the samples were collected (Santa Catarina-SC, Paraná-PR, São Paulo-SP, Rio de Janeiro-RJ, Espírito Santo-ES, Minas Gerais-MG, Goiás-GO, Pernambuco-PE). The solid line represents the Equator. Starred and dotted lines mark Intertropical Convergence Zone (ITCZ). Map generated with R 3.2.3 (http://www.R-project.org/) and edited with Gimp 2.8 (https://www.gimp.org).

The results of the radiocarbon isotopic ratios for each of the mollusk shells along with the collecting years and locations are presented in Table 1. We have also included the results from 3 *Thaumastus achilles* samples collected alive in 2013 AD, from the work of Carvalho *et al.*^22^. Sample codes from the USP zoology museum collection are presented, as well as lab codes for each radiocarbon age measured. Radiocarbon determinations for four of the samples were duplicated at the Fluminense Federal and Oxford universities’ laboratories. Location refers to the Brazilian states as shown in the map in Fig. 2.

The values are expressed in Fraction Modern, i.e. the radiocarbon isotopic ratio corrected for isotopic fractionation and divided by the hypothetic isotopic ratio of the atmospheric reservoir in 1950 AD^36^.

**Figure 3:**
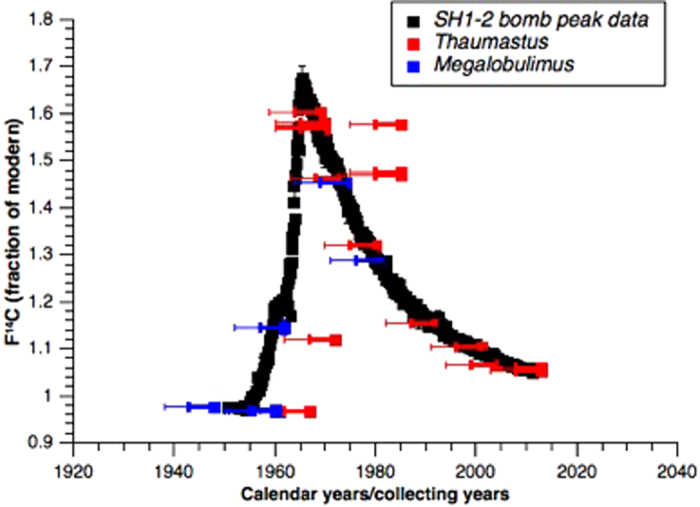
Red: *Thaumastus sp*. Blue: *Megalobulimus sp.* Fraction of modern carbon of the measured land snails x collecting years (a negative error bar was applied to estimate the longevity of individuals up to 5 or 10 years); Black: post-bomb atmospheric SH1-2 calibration curve^35^.

Figure 3 shows the results of Fraction Modern for the land snails versus their collecting dates, along with the SH1-2 bomb peak curve^35^. Since the shell grows during the animal’s life, its composition may reflect the radiocarbon concentration of the atmosphere in any of the years it was alive. Although gastropod shells grow in a spiral pattern it is not straightforward that growth bands would contain only carbon from specific years since there is evidence that broken shells can regenerate and will therefore contain carbon from any of which years. A 10 years old gastropod sample collected in 1970, for instance, is likely to contain carbon incorporated in 1960, before the peak, when atmospheric levels were lower than those of 1970. Consistently, the same could happen for samples collected in 1980, as they enclose carbon from 1970, when atmospheric levels were higher than those of 1980. Since the measured samples are small compared to the size of the shell, there is also the possibility of the sampling process selecting carbon from the year of death of the animal, what would lead to the actual atmospheric levels at such year. For this reason, an asymmetric error bar was used, in order to account for the animals’ lifespan.

## Discussion

From the Fraction modern results presented in Table 1 it is possible to evaluate the consistency between the two laboratories. Results are statistically similar for the four compared samples, which indicates that the different preparation methods (see methods section) give equivalent results.

In Fig. 3 it can be observed that, for both *Thaumastus* and *Megalobulimus* genera, most of the dating results follow the SH1-2 bomb ^14^C concentration in respective collecting years within less than 5 years range. This implies that these terrestrial snails can correctly record the radiocarbon atmospheric concentration over time.

Since both genera of terrestrial mollusks were found to be in equilibrium with the atmospheric carbon reservoir during their lives, they constitute a satisfactory alternative to the use of charcoal in chronological studies within archaeological sites. According to Pigati, Rech & Nekola^21^, carbonate in gastropod shells can have four different sources: atmospheric CO_2_ (by respiration), food (plants and general organic detritus), water (from dew, soil moisture, standing water and precipitation) and carbonate rocks (scraping limestone, dolomite or soil carbonate). Amounts of each source contribution are variable and depend on species and habitat. Amongst these sources, incorporation from limestone and other carbonate rocks presents the most significant problem to using shells as a proxy for atmospheric carbon^21^.

According to Wilbur^37^ bicarbonate in the extrapallial fluid is originated in the hemolymph or by direct diffusion of CO_2_ from the environment^13^ and is then deposited in the shell carbonate. Rubin, Likins & Berry^12^ claim that major inputs of limestone-derived carbon come from ingestion, followed by dissolution in the gut and diffusion into the hemolymph, or from uptake of limestone dissolved in secretions through the foot^17^,^38^. Considering the observed results, we excluded the possibility of the incorporation of old carbon by most of the studied herbivorous terrestrial snails of *Thaumastus* and *Megalobulimus*^22^,^39^. According to the distribution of the karst regions in Brazil^40^, most of the studied sites present potential availability of carbonate rocks. Moreover, for regions as São Domingos, in the state of Goiás, where samples were collected from inside a cave with great amount of limestone, or important areas of carbonate rocks exploitation, as Aracruz and Baixo Gandú, in the state of Espírito Santo, the studied snails are not affected by the dead carbon effect for reasons that remain unexplained. According to Pigati, Rech & Nekola^21^ the need for calcium would push larger snails to obtain it from rocks as opposed to smaller taxa, which would fulfil their needs with a regular diet (plants, detritus and water). If this was the case for the studied taxa, especially for the very large snails from the *Megalobulimus* genera, dietary contribution to the shell composition would be negligible, since none of the studied snails did incorporate dead carbon.

Four of the analysed *Thaumastus sp.* samples are between 10 and 15 years far from the curve (MZUSP 111243, MZUSP 104511 and 2 shells from MZUSP 62044). Samples MZUSP 111243 and MZUSP 104511 present values lower than the corresponding collecting years, 1972 and 1967, respectively. This could be explained by incorporation of dead carbon by the *Thaumastus taunaisii* snail collected in Iporanga, SP, a recognized area of limestone. However, in the case of the *Thaumastus achilles* snail, collected at Marataizes, ES, this explanation is not so straightforward. Although the Espírito Santo state has important exploitation sites for ornamental stones production, the southern coast is characterized by Quaternary sand deposits intercalated with cliffs of the Tertiary Barreiras formation with no significant limestone presence^41^.

Concerning the two individual samples from MZUSP 62044 batch, the results are higher than the atmospheric concentration in 1985. This suggests that the reason for the lag in dates for all four samples which fall away from the bomb curve is due to lifespan rather than the incorporation of fossil carbon through scraping. Hence, it is very likely that they were collected (or died) a few years earlier than the date this specific samples were handed to the museum by fisherman (see methods section). Another possible reason for discrepancy for these four samples could be the lifespan of terrestrial mollusks, as previously discussed.

There is not much in literature on the longevity of such animals^42^,^43^,^44^. Moreover, the diversity of species is great and the lifespans depends on many factors. In a survey of the available literature, Heller^42^ has revealed longevity records of 75 species from 57 different genera from an overall estimate of 1700 existing genera. According to this author, lifespans of terrestrial gastropods usually range from several months to 19 years. A study on the species *Placostylus hongii* (Pulmonata: Bulimulidae) estimates a lifespan of at least 10 years^43^. Large snails, such as some species of *Megalobulimus*, are believed to live for decades. Recent studies on specific species reveal lifespans of about 30 years for *Megalobulimus paranaguensis*^44^ or even up to 88 years for *Megalobulimus intertextus*^45^.

The taxonomy of such land snails is still confused, the number of species is very likely underestimated and, if there is poor information in relation to the species themselves, a worse scenario can be expected in relation to the remaining biological information. It is expected that the ongoing analysis of several samples from the same shell will help to solve this question and maybe to understand whether there is a pattern of growth that could be related to age. Further work is also in progress in order to estimate more precise lifespans for the studied animals.

For archaeological studies, a lifespan of 20 years or less would not represent a problem. It is less than the typical uncertainties from radiocarbon determinations and it is less than the typical lifespan of a short lived tree species. Therefore, charcoal samples would provide similar or even larger uncertainties if lifespan was to be considered. A terrestrial mollusk based chronology would then provide results as accurate as charcoal samples would, presenting an option for charcoal samples from long-lived trees, making it possible to avoid the old-wood effect. Furthermore, the conclusions drawn here allow chronological studies to be performed in sites with either none or non-contextualized charcoal.

For the *Thaumastus taunaisii* and the *Thaumastus achilles* taxa, more studies are needed to explain the observed discrepancy. Measurements of present day specimens from such species in areas of limestone would help to confirm whether the differences in concentration are due to the animal’s lifespan or to the incorporation of dead carbon. Either way, for archaeological samples collected in areas without limestone, the method would still be valid. The conclusions of this study are valuable in the sense that they expand the variety of possible studies on Brazilian shellmounds and other archaeological sites in South America, where the presence of the studied genera have been reported.

In the work of Carvalho *et al.*^22^ *Thaumastus achilles* shells and charcoal samples from the Manitiba shellmound, in Saquarema, RJ, were found to have similar radiocarbon concentration, with conventional ages ranging from 3659 ± 25 to 3748 ± 38 yr BP for terrestrial snails and from 3598 ± 46 to 3747 ± 22 yr BP for charcoal. Ongoing work on archaeological *Megalobulimus* specimens from the Usiminas shellmound, in Ilha de Cabo Frio, RJ, also seem to corroborate our results. On the other hand, none of these areas have carbonate rocks, what would limit the validity of such works for non-karstic areas.

Another issue concerning mollusk shells, either for marine or terrestrial organisms, could be the possibility of recrystallization since it has been observed that for old samples, aragonite can transform into calcite, allowing exogenous carbon atoms to be incorporated^20^,^46^. In this case, monitoring with X-ray diffraction, for example, can give information regarding the aragonite/calcite ratios. Even though extra care is required, carbonate samples are the most straightforward, cheaper and less time consuming to chemically prepare.

## Methods

### Sample selection

For this study we have analyzed 12 *Thaumastus* and 6 *Megalobulimus* selected from the MZUSP collection. The animals were sampled by researchers over the last decades for zoological purposes across several Brazilian States, comprising Cerrado and Atlantic forest biomes, in regions with and without limestone. Our aim was to relate the collection dates of the animals to the radiocarbon bomb peak based on their radiocarbon ratios, provided that the samples were collected alive by zoologists and the collection dates were accurately recorded. Since terrestrial snails’ shells could contain carbon from any year during the animal’s life, we took into account a negative error bar considering an estimate longevity. Two of the samples (MZUSP 62044) were handed to the museum by fishermen, so the collection date should be understood as a limit year, since they could have been collected dead or stored for an unknown period of time.

### Sample preparation and measurement

The shells had their radiocarbon content measured at either the Radiocarbon Laboratory of the Fluminense Federal University (LAC-UFF), in Brazil, or at the Oxford Radiocarbon Accelerator Unit (ORAU), United Kingdom. Four of the studied samples were prepared and measured in both laboratories to check for consistency.

The sample preparation followed standard procedures in each laboratory^47^,^48^. From each shell a sample of approximately 40 mg was collected by cutting the shell with a diamond cutter. At ORAU samples were soaked in Feigl’s solution to indicate the presence of calcite from recrystallization. The outer layer, which could have been contaminated, was removed with a sand blaster at ORAU while at LAC-UFF it was done by etching with hydrochloric acid. In both laboratories, all the samples were then hydrolyzed in phosphoric acid and the carbon dioxide purified and graphitized. At LAC-UFF graphitization is performed in independently sealed pyrex tubes containing titanium hydride and zinc at the bottom and an inner tube with iron. The tubes are baked at 520 °C for 7 h^47^. At ORAU the graphitization takes place in a double fold tube filled with hydrogen gas. The side of the tube containing iron is heated to 560 °C for 6 h^49^. At ORAU the samples were measured in a High Voltage 2 MV accelerator^50^ and at LAC-UFF the samples were measured in a single stage 250 kV AMS system built by National Electrostatics Corporation. The measurements were corrected for isotopic fractionation using stable isotopes ratios measured simultaneously in the accelerator.

## Additional Information

**How to cite this article**: Macario, K.D. *et al.* The use of the terrestrial snails of the genera *Megalobulimus and Thaumastus* as representatives of the atmospheric carbon reservoir. *Sci. Rep.* **6**, 27395; doi: 10.1038/srep27395 (2016).

## Figures and Tables

**Figure 1:**
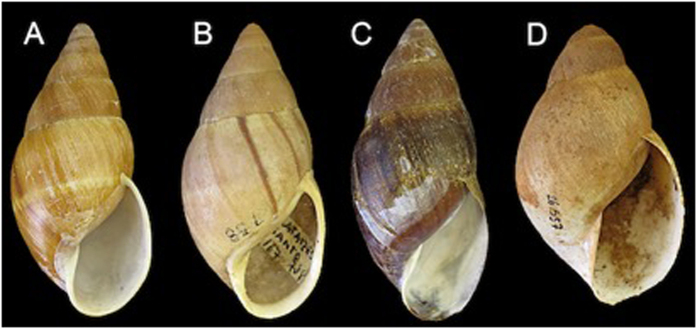
Apertural view of used species (measurements made with a digital caliper). (**A**) *Thaumastus taunaisii* MZUSP 104511 (length = 63.4 mm); (**B**) *Thaumastus achilles* MZUSP 111243 (length = 50.9 mm); (**C**) *Thaumastus magnificus* MZUSP 72411 (length = 72.9 mm); (**D**) *Megalobulimus sp.* MZUSP 26557 (length = 56.0 mm).

**Table 1:** Collecting years and Fraction modern carbon obtained from the set of analysed samples.

Collecting year (AD)	Fraction modern	Genus/Species	LAB code	Sample code	Location	Latitude	Longitude
1948	0.9757 ± 0.0028	*Megalobulimus granulosus*	LACUFF150209	MZUSP 16581	Piçarras, SC	26° 45′50″S	48° 40′18″W
1960	0.9702 ± 0.0032	*Megalobulimus sanctipauli*	OxA-31912	MZUSP 29296	Luiz Alves, SC	26° 43′14″S	48° 55′58″W
	0.9767 ± 0.0039		LACUFF150206				
1961	0.9681 ± 0.0032	*Megalobulimus sanctipouli*	LACUFF 150207	MZUSP 29296	Luiz Alves, SC	26° 43′14″S	48° 55′58″W
1962	1.1454 ± 0.0156	*Megalobulimus sp.*	LACUFF 150329	MZUSP 29557	São Domingos, GO	13° 37′42″S	46° 19′27″W
1967	0.9653 ± 0.0047**	*Thaumastus achilles*	LACUFF 150334	MZUSP 111243	Marataizes, ES	21° 02′36″S	40° 49′28″W
1969	1.6041 ± 0.0092	*Thaumastus taunaisii*	LACUFF 150330	MZUSP 111230	Igaratá, SP	23° 12′ 16” S	46° 09′ 22” W
1970	1.5752 ± 0.0035	*Thaumastus sp.*	OxA-31909	MZUSP 26762	Baixo Gandú, ES	19° 31′08″S	41° 00′57″W
	1.5694 ± 0.0037		LACUFF150204				
1970	1.5707 ± 0.0034	*Thaumastus sp.*	OxA-31910	MZUSP 26762	Baixo Gandú, ES	19° 31′08″S	41° 00′57″W
	1.5790 ± 0.0044		LACUFF150205				
1972	1.1207 ± 0.0055^**^	*Thaumastus taunaisii*	LACUFF 150333	MZUSP 104511	Iporanga, SP	24° 35′08″S	48° 35′35″W
1973	1.4644 ± 0.0065	*Thaumastus magnificus*	LACUFF 150336	MZUSP 70654	Lima Duarte, MG	21° 50′33″S	43° 47′35″W
1974	1.4539 ± 0.0035	*Megalobulimus terrestris*	LACUFF 150210	MZUSP 29323	Caruarú, PE	08° 17′00″S	35° 58′34″W
1980	1.3193 ± 0.0061	*Thaumastus sp.*	LACUFF 150337	MZUSP 29556	Piraquara, PR	25° 26′38″S	49^o^03′44″W
1981	1.2886 ± 0.0031	*Megalobulimus terrestris*	OxA-31911	MZUSP 28900	Orobó, PE	07° 44′42″S	35° 36′08″W
1985	1.5788 ± 0.0036**	*Thaumastus sp.*	OxA-31907	MZUSP 62044	Baixo Gandú, ES	19° 31′08″S	41° 00′57″W
1985	1.4697 ± 0.0034**	*Thaumastus sp.*	OxA-31908	MZUSP 62044	Baixo Gandú, ES	19° 31′08″S	41° 00′57″W
	1.4761 ± 0.0038**		LACUFF150203				
1992	1.1542 ± 0.0055	*Thaumastus sp.*	LACUFF 150335	MZUSP 30461	Aracruz, ES	19° 49′13″S	40° 16′24″W
2001	1.1032 ± 0.0066	*Thaumastus sp.*	LACUFF 150331	MZUSP 33039	Praia Grande, SP	24° 00′21″S	46° 24′10″W
2004	1.0665 ± 0.0069	*Thaumastus magnificus*	LACUFF 150332	MZUSP 72411	Macaé, RJ	22° 22′15″S	41° 47′13″W
2013	1.0514 ± 0.0060	*Thaumastus achilles*	LACUFF 140577	MT1*	Cabo Frio, RJ	22° 52′46″S	42° 01′07″W
2013	1.0593 ± 0.0060	*Thaumastus achilles*	LACUFF 140578	MT2*	Cabo Frio, RJ	22° 52′46″S	42° 01′07″W
2013	1.0539 ± 0.0060	*Thaumastus achilles*	LACUFF 140579	MT3*	Cabo Frio, RJ	22° 52′46″S	42° 01′07″W

^*^Results from ref. 22.

^**^Results that plot further than 10 years from the bomb curve.
